# Evaluation of Infection-Related Hospitalizations and Drug Prescriptions Among Sepsis Survivors in Germany

**DOI:** 10.1001/jamanetworkopen.2022.20945

**Published:** 2022-07-08

**Authors:** Carolin Fleischmann-Struzek, Bianka Ditscheid, Josephine Storch, Norman Rose, Melissa Spoden, Christiane S. Hartog, Antje Freytag

**Affiliations:** 1 Institute of Infectious Diseases and Infection Control, Jena University Hospital, Jena, Germany; 2 Center for Sepsis Control and Care, Jena University Hospital/Friedrich Schiller University Jena, Jena, Germany; 3 Institute of General Practice and Family Medicine, Jena University Hospital, Jena, Germany; 4 Research Institute of the Local Health Care Funds (AOK), Berlin, Germany; 5 Department of Anesthesiology and Operative Intensive Care Medicine, Charité Universitätsmedizin Berlin, Berlin, Germany; 6 Klinik Bavaria, Kreischa, Germany

## Abstract

This cohort study assesses infection-related hospitalizations and outpatient drug prescriptions among sepsis survivors in Germany and compares changes in hospitalization and prescription rates before and after sepsis occurrence.

## Introduction

New or recurrent infections and sepsis are leading causes of rehospitalization after sepsis.^1^ Although persistent immunosuppression after sepsis is considered a causative factor, patient-inherent risk factors may also contribute to increased risk of recurrent severe infections.^2^ The burden of infection in these patients presepsis is unknown. We analyzed the change in infection-related hospitalizations and outpatient drug prescriptions presepsis vs postsepsis.

## Methods

This retrospective cohort study was based on health claims data of AOK, a statutory health insurance provider in Germany, for the years 2011 to 2015. The study was approved by the Jena University Hospital Institutional Review Board; informed consent was waived owing to deidentified patient data. This study followed followed the STROBE reporting guideline.

Inpatient sepsis cases were identified by *International Statistical Classification of Diseases and Related Health Problems, Tenth Revision, German Modification* (*ICD-10-GM*) codes between January 1, 2013, and December 31, 2014, among AOK beneficiaries who were older than 15 years and had no sepsis in the 24 months before index hospitalization. We analyzed hospitalizations and drug prescriptions in the 12 months presepsis and postsepsis among sepsis survivors. Hospitalizations were classified as infection- or sepsis-related according to requisite *ICD-10-GM* hospital discharge diagnoses.^3^ Intensive care unit (ICU) treatment was identified by Operation and Procedure Classification System codes for intensive care complex treatment (8-980, 8-98f, 8-98d, 8-98c). We analyzed total drug prescriptions and prescriptions of anti-infectives according to Anatomic Therapeutical Chemical codes (J01, J02, J04A, J05, A07AA, P01AB). Presepsis and postsepsis outcomes were compared using a 2-sided McNemar χ^2^ test; statistical significance was set at α = .05. Statistical analyses were conducted using SAS Enterprise Guide, version 7.1 (SAS Institute Inc).

## Results

Among 23 million AOK beneficiaries, we identified 159 684 sepsis patients, 116 507 of whom survived hospitalization. The mean (SD) age was 73.0 (13.3) years; 52.1% were men and 47.9% were women. Among the survivors, 32.5% had severe sepsis, 27.7% were treated in an ICU, and 7.4% had no preexisting impairments. In the 12 months postsepsis, 66.8% of survivors were rehospitalized and 45.0% were rehospitalized with infection (67.4% of all rehospitalizations). Among all survivors, 11.9% were rehospitalized for recurrent sepsis, 25.9% of whom were admitted to an ICU; 56.6% of sepsis survivors received anti-infective treatment in an outpatient setting.

Although hospitalization rates increased by 3.4% from 63.4% in the 12 months presepsis to 66.8% in the 12 months postsepsis (*P* < .001), the proportion of patients with infection-related hospitalizations increased by 9.6% (presepsis, 35.4% vs postsepsis, 45.0%; *P* < .001). The proportion of patients with device-related infections nearly doubled from presepsis to postsepsis (Figure). Total outpatient drug prescriptions decreased (97.5% vs 94.1%; *P* < .001), but the proportion of patients with at least 1 anti-infective prescription increased by 4.0% (52.6% vs 56.6%; *P* < .001). The increase in hospitalization and infection-related hospitalization rates was highest in patients without preexisting medical, cognitive, or psychological impairments (hospitalization rate, from 22.5% to 54.6% [a 32.1% increase]; infection-related hospitalization rate, from 6.4% to 31.4% [a 25.0% increase]) (Table). Prescriptions of anti-infectives increased consistently across subgroups and were most prominent in the subgroup of survivors without preexisting impairments (16.7%).

**Figure.**
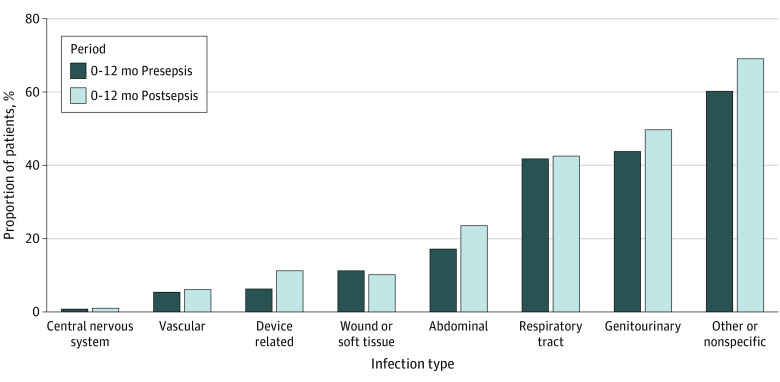
Comparison of Presepsis and Postsepsis Source of Infection Among Patients With Infection-Related Hospitalization

**Table.** Comparison of Hospitalizations, Infection-Related Hospitalizations, and Drug Prescriptions Among Patients Before and After Sepsis Hospitalization

Variable^a^	Patient group								
	Total	Patients with ≥1 hospitalizations		Patients with infection-related hospitalizations		Patients with ≥1 drug prescriptions		Patients with anti-infective prescriptions	
	No.	No. (%)	*P* value	No. (%)	*P* value	No. (%)	*P* value	No. (%)	*P* value
Sepsis									
Severe									
Presepsis	37 840	23 800 (62.9)	<.001	13 474 (35.6)	<.001	36 747 (97.1)	<.001	19 104 (50.5)	<.001
Postsepsis	37 840	25 409 (67.1)		17 757 (46.9)		34 472 (91.1)		20 074 (53.1)	
Nonsevere									
Presepsis	78 667	50 082 (63.7)	<.001	27 785 (35.3)	<.001	76 805 (97.6)	<.001	42 121 (53.5)	<.001
Postsepsis	78 667	52 433 (66.6)		34 688 (44.1)		75 196 (95.6)		45 908 (58.4)	
ICU admission									
Yes									
Presepsis	32 238	20 305 (63.0)	<.001	10 970 (34.0)	<.001	30 980 (96.1)	<.001	15 354 (47.6)	<.001
Postsepsis	32 238	22 407 (69.5)		15 426 (47.8)		29 371 (91.1)		16 440 (51.0)	
No									
Presepsis	84 269	53 577 (63.6)	<.001	30 289 (35.9)	<.001	82 572 (98.0)	<.001	45 871 (54.4)	<.001
Postsepsis	84 269	55 435 (65.8)		37 019 (43.9)		80 297 (95.3)		49 542 (58.8)	
Prior impairment									
Yes									
Presepsis	107 885	71 943 (66.7)	<.001	40 710 (37.7)	<.001	106 943 (99.1)	<.001	58 247 (54.0)	<.001
Postsepsis	107 885	73 137 (67.8)		49 735 (46.1)		101 708 (94.3)		61 566 (57.1)	
No									
Presepsis	8622	1939 (22.5)	<.001	549 (6.4)	<.001	6609 (76.6)	<.001	2978 (34.5)	<.001
Postsepsis	8622	4705 (54.6)		2710 (31.4)		7960 (92.3)		4416 (51.2)	
Age, y									
<40									
Presepsis	2649	1392 (52.5)	<.001	703 (26.5)	<.001	2381 (89.9)	<.001	1610 (60.8)	<.001
Postsepsis	2649	1560 (58.9)		903 (34.1)		2461 (92.9)		1734 (65.4)	
40-65									
Presepsis	25 860	15 928 (61.6)	<.001	8406 (32.5)	<.001	24 472 (94.6)	.58	14 214 (55.0)	<.001
Postsepsis	25 860	17 703 (68.4)		11 272 (43.6)		24 445 (94.5)		15 133 (58.5)	
66-80									
Presepsis	51 787	33 889 (65.4)	<.001	19 076 (36.8)	<.001	50 831 (98.2)	<.001	27 158 (52.4)	<.001
Postsepsis	51 787	35 774 (69.1)		24 382 (47.1)		48 726 (94.1)		29 540 (57.0)	
>80									
Presepsis	36 211	22 673 (62.6)	.29	13 074 (36.1)	<.001	35 868 (99.0)	<.001	18 243 (50.4)	<.001
Postsepsis	36 211	22 805 (63.0)		15 888 (43.9)		34 036 (94.0)		19 575 (54.0)	

^a^ All presepsis and postsepsis periods are 12 months.

## Discussion

This study found that infection-related hospitalizations affected 2 of every 3 sepsis survivors in Germany. Although hospitalizations among our study cohort increased by 9.6% postsepsis, more than half of these patients had already contracted infectious diseases requiring hospitalization prior to sepsis. This finding suggests that many sepsis patients are at risk for severe infections presepsis—presumably owing to preexisting immune dysfunction—and that septic insults may exacerbate their risk of developing severe infections and recurrent sepsis.^4^ Furthermore, patients without prior impairments and low rates of presepsis infection–related hospitalizations and drug prescriptions had these rates increase substantially postsepsis.

This study has some limitations. First, the validity of health claims diagnoses relies on coding quality. Second, subgroup comparisons should be interpreted within the context of differential mortality rates among survivors. Regardless, our findings highlight the need for preventive measures—particularly vaccinations and programs to prevent device-related infections—as well as early recognition and education regarding symptoms among all sepsis survivors and at-risk patients in the general population.
